# Food Provision at the Olympic Games in the New Millennium: A Meta-narrative Review

**DOI:** 10.1186/s40798-023-00567-7

**Published:** 2023-04-21

**Authors:** Fiona E. Pelly, Judith Tweedie, Helen O’Connor

**Affiliations:** 1 School of Health, University of the Sunshine Coast, Sippy Downs, QLD Australia; 2 Faculty of Medicine and Health, Sydney School of Health Sciences, The University of Sydney, Camperdown, Australia

**Keywords:** Meta-narrative, Athletes, Competition, Food provision, Olympic, Food safety, Environmental sustainability

## Abstract

**Background and objective:**

The objective of this meta-narrative review was to identify, organise and map the literature on food provision and nutrition support at the summer and winter Olympic and Paralympic Games (OPG)  and similar major competition events over the past 21 years. This builds on a comprehensive update of a previous historical review of the evolution of food provision at the summer Olympic Games up until 2000 and considers contemporary issues such as the global pandemic and sustainability goals.

**Methods:**

A range of sources included primary research and review articles, edited book chapters, theses, conference papers or abstracts, International Olympic Committee reports, Organising Committees’ food vision and post-Games reports, independent professional reports, and media and periodicals including magazines and trade journals. The search strategy included four steps: a database search that complied with Preferred Reporting Items for Systematic Reviews and Meta-Analyses extension for Scoping Reviews criteria, a search of the Olympic Studies Centre, a review of reference lists for unpublished sources, and a Google search for additional media reports. The researchers followed an iterative process where emerging narratives were discussed, recorded and refined as data were extracted.

**Results:**

The data from 229 records were extracted into a spreadsheet and grouped according to the type of evidence and specific event, then presented chronologically to give a perspective on the development of food provision and nutrition support. Eleven narratives emerged from the data extraction: ‘description of meals, menus and food’, ‘vision of the food provision’, ‘food safety’, ‘catering company involvement’, ‘sponsorship or contracts with food companies’, ‘athlete perspective’, ‘stakeholder perspective’, ‘athlete food intake,’ ‘nutrition input in food provision’, ‘food environment’ and ‘sustainability’.

**Conclusion:**

Results suggest that athletes' dining expectations, organising committee budgets, expert input and current global trends have led to food delivery changes. The OPG food environment has the capacity to positively influence the dietary choices of athletes and teams, while evolving to meet contemporary global challenges such as COVID-19 and sustainability targets.

**Supplementary Information:**

The online version contains supplementary material available at 10.1186/s40798-023-00567-7.

## Key Points

- This meta-narrative review provides a comprehensive overview of food provision and at the Olympic and Paralympic Games (and similar scoped major competition events) over the past 21 years (since 2000) and discusses future challenges. Information sources included both grey literature (media, periodicals), official reports and evidence-based studies, reviews, book chapters and theses.
- Eleven narratives emerged from the data extraction which were synthesised into four overarching themes: (1) Scope, experience of caterers, role of sponsorship and outcomes of food provision; (2) Food safety, security and risk of illness (including COVID-19); (3) The role of nutrition in food provision (including dietary trends and variability in nutrition servicing of athletes); and (4) The increased global focus on sustainability in relation to large scale competition events.
- The food environment at the Olympic and Paralympic Games is an example of ongoing excellence in food provision through the incorporation of sports nutrition innovation and science to ensure the provision of a safe and sustainable food supply that can educate on health and performance of athletes and showcase the value and role that food has in society.

## Introduction

The primary purpose of food provision at the Olympic and Paralympic Games (OPG) is to ensure the availability of safe, appealing food suitable for athletes, support staff and officials of various ages and cultural backgrounds. From a sports nutrition perspective, suitable foods must be provided to allow optimum performance and recovery for athletes from a wide array of sports with varying nutritional demands [1]. The ‘performance-based menu’ is informed by the scientific literature and position stands, which make recommendations on nutrition for athletic performance [1]. To date, the majority of research relevant to the OPG on the topic of food and diet has focused on the dietary intake or eating behaviours of athletes with an outcome of sports performance with less information about the food provided and more broadly the overall food environment. Yet, the influence of the food environment is paramount to food choice in athletes [2–4]. The food environment encompasses, for example, the availability and accessibility of food, characteristics of the eating environment, food promotion, and cost of food [2, 3]. Thus, the food environment at the OPG is an important driver for not only the performance of athletes but the health of all attending.

An historical review [5] from 2011 summarised the food provision at the summer Olympic Games (OG) from Olympic reports and existing scientific literature. It is apparent from the review that athletes have been provided with some form of food since the modern OG in 1896, where there are reports that competitors in the marathon were provided with olives, eggs, cheese, milk and oranges before they raced [6]. This review describes how athletes typically used hotel dining rooms or local restaurants, although some teams travelled with personal chefs [5]. With the advent of the Olympic village in 1932 [7], there was expansion in catering for athletes, although food provision was basic and commonly altered at the request of individual nations [5]. By 1948, nations were allocated housing based on their ‘feeding requirements’ (i.e. French, Scandinavian, South American and Spanish, North American, Central European and specialised nations), and a total of four catering contractors and 25 kitchens were assigned to the various accommodation venues [8]. Little arrangement was made for catering at venues. The first ‘almost continuous’ service [8] resulted as a consequence of specific meal periods not being able to align with competition schedules.

Post this time, there were developments in food provision, including the commencement of the Paralympic Games for athletes with physical disabilities in 1960 [9], introduction of a unified menu in 1972 [10], nutrition labelling in 1992 [11] and a nutrition 'kiosk' introduced in 1996 [12], but there was little evidence to evaluate the suitability and impact of this service until the Sydney 2000 OG. Other historical reports, including grey literature, were scant prior to 2000 and focused on the culinary or hospitality aspects [13–29] of the food provision. There was minimal focus on the Paralympic or winter Olympic Games. There was anecdotal feedback from the 1996 OG in Atlanta suggesting a higher than anticipated demand for traditional foods from African, Asian and Eastern European nations [30] representing a change in expectations by those attending.

Historical evidence, more established sports nutrition guidelines and what seemed to be an emerging change in village dining expectations helped to shape a project that was implemented at the Sydney 2000 OG [30, 31]. The implementation of the project focused on the total food environment and facilitated the significant involvement of sports, clinical and food service dietitians in menu development and food service operation for village dining for the first time [32]. This incorporated a review and dietary analysis of the food provided in the main dining hall, point of choice nutrition labelling, development of a website advertising the menu prior to the Games, and training of catering staff. Extended nutrition kiosk services were offered to athletes, officials and guests by sports and clinical dietitians. Experienced sports and clinical dietitians mentored local students, building capacity across the sports nutrition profession. While the catering and nutrition support for the Sydney 2000 OG was reported to be highly successful [30], the impact of this approach has not been thoroughly explored, and the long-term outcomes have not been evaluated.

Over the past 20 years since the 2000 OG, significant scientific advances have been made in understanding the benefits of tailoring dietary intake to optimise health and sports performance [1, 33]. There is also increased demand in therapeutic dietary needs (such as food allergies or intolerances) [34, 35], diets to manage gastrointestinal distress [36] and a wide range of clinical conditions where diet is central to clinical management (e.g., diabetes, coeliac or cardiovascular disease, inflammatory or irritable bowel syndrome) [35]. There has also been substantial growth in the Paralympic Games from 400 to over 4000 participants and 23 to 162 countries [37]. As this event immediately follows the Olympic Games in the same village environment and athletes can have specific needs [38], appropriate food provision is important. This is distinct from the Commonwealth Games where events for athletes with a disability are integrated into the competition.

Concern about environmental sustainability has also increased on a global scale with the release of the EAT-Lancet Commission on healthy diets from sustainable food systems [39], which advocates the need for sustainable food systems that not only provide health benefits but also sustain a healthy planet. While foodborne illness is of concern at any large-scale event, the COVID-19 pandemic is a new area of concern in relation to the transmission of viruses at major competition events.

Thus the objective of this review was to identify, organise and map the literature on food provision at the summer and winter OPG (and similar scoped mega and major competition events [40] including the Commonwealth Games, Pan American Games, Asian Games and Universiade) over the past 21 years, report on past and current outcomes, and discuss future challenges. The specific questions we set to answer are:

- What aspects of food provision have been described in the literature over the past 21 years?
- What empirical evidence has been published in relation to food provision at these events?
- What are the outcomes from the evidence that has been reported in relation to each event? (e.g., types of meals and menus, uptake of food, stakeholder opinion of the food provision, nutrition servicing, environmental sustainability and wastage, food safety, other aspects relevant to the food environment)
- What additional documents exist that relate to food provision at these events, and what insights can be gained by combining these different sources of information?
- What are the implications for practice and future policy?

This study presents a systematic meta-narrative review of the past 21 years of food provision at major competition events including both summer and winter Olympic Games and other relevant competition events. This study will build on the previous narrative review by Pelly et al. [5], by including not only scientific evidence but other sources of evidence to describe comprehensively the literature on food provision from 2000 to the present day, the narratives and multiple perspectives emerging from the literature, and to identify future challenges and directions in the context of a changing food environment.

## Methods

To examine this topic, we used an adaptation of a meta-narrative approach to this review [41]. This method allows for heterogeneity and complexity of the various sources of evidence and thus a richer understanding of the topic area [41]. The meta-narrative review was guided by six principles of pragmatism (inclusion of what makes sense), pluralism (multiple perspectives), historicity (evolution over time), contestation (conflicting data examined to generate higher-order insights), reflexivity (reflect on emerging findings) and peer review [41]. This approach allows for varied stakeholder perspectives and the inclusion of media and descriptive reports as well as empirical evidence. A meta-narrative approach fits with this topic due to the diversity of perspectives on food provision at major events and is a strategy to help policy makers with guided decisions. This review follows the RAMESES (Realist And Meta-narrative Evidence Syntheses: Evolving Standards) publication standards [42]. The six guiding principles were followed as outlined below: (1) Pragmatism: the review was guided by what the authors felt was the most useful information for the intended audience of researchers, practitioners and policymakers. (2) Pluralism: the topic of food provision and at mega and major events was reviewed and considered a range of outcomes reported from both grey and peer review literature to gain insights from different sources with multiple stakeholder perspectives. (3) Historicity: the literature was reviewed over a 21-year period from the 2000 Sydney OG to pre the 2022 Tokyo OPG to determine how food provision and nutrition support has been researched and presented over time. (4) Contestation: conflicting data from the different literature sources were examined to gain insights into how food provision at mega games is examined. (5) Reflexivity: when performing the review, each of the members of the review team took time to reflect on the findings, individually and as part of the review team. (6) Peer review: informal discussion of the findings occurred through discussion with research assistants and experts through the data extraction and interpretation phase.

### Search Strategy

In planning the review, the initial scoping of the literature was conducted by the first author who had prior knowledge of the topic and range of evidence. The planning phase included deciding the sources of evidence that would be included to ensure different perspectives were captured. The authors identified ten types of evidence that would adequately cover the 21-year period of time: five evidence-based sources (primary research studies, review articles, edited book chapters, theses and conference papers or abstracts), four types of reports (International Olympic Committee reports, organising committees’ food vision and post-Games reports, and independent professional reports) and two types of grey literature (media and periodicals including magazines and trade journals). This informed the search strategy, which included a four-step process as follows:

1. A systematic approach to searching standard databases was undertaken with a clearly defined objective (as mentioned previously), inclusion criteria and specific search terms as per the Preferred Reporting Items for Systematic Reviews and Meta-Analyses extension for Scoping Reviews (PRISMA-ScR) [43] criteria. Standard databases searched were Scopus, Web of Science PubMED, SPORTDiscus, and ProQuest Dissertations and Theses. The following search terms were used to identify articles relevant to specific events: **‘**Olympic*’,‘Olympiad’, ‘Paralympic’, ‘Commonwealth Games’, ‘Universiade’, ‘Pan American games’, ‘Asian games’, ‘mega-event’ and ‘sporting event’. These were then combined with the following search terms using BOOLEAN operators ‘catering’, ‘foodservice’, ‘food provision’, ‘food*’, ‘menu*’, ‘nutrition’, ‘HACCP’, ‘dining’, ‘feeding’ or ‘sustainability and food’. Empirical evidence and review articles in peer review journals, abstract and conference proceedings, edited book chapters, reports, theses, periodicals (including trade magazines) and media reports were included for review. Articles that reported on dietary intake or supplement use of Olympic or other athletes that were not specifically relevant to food provision at the event were excluded, as were public health reports on foodborne outbreaks unrelated to the village or venue food provision and studies conducted at smaller scale competition events. Any literature that was not available in English was excluded. The search was conducted in June 2020, updated in June 2021 and repeated in May 2022 to capture any additional evidence. The dates for inclusion were from January 2000 up until 31st July 2021. The rationale for the end date was the commencement of the 2021 Tokyo OPG.
2. A search of the Olympic Studies Centre (https://library.olympic.org/Default/accueil.aspx) was conducted to find official reports by organising committees of past OPG, relevant IOC official documents, Olympic periodicals and reports relevant to food provision at mega events.
3. Review of the reference lists and bibliographies of articles from the initial search for systematic snowball sampling was also conducted to find additional evidence or unpublished/ unofficial reports.
4. To find media articles beyond those identified through the initial search, Olympic, Olympic village, Commonwealth Games, Universiade, Pan American and Asian Games combined with foodservice and catering terms were entered into Google search. As this did not result in any relevant information, the name of the specific event along with foodservice and catering terms was entered and the first 20 pages were searched for relevant records. Each result from the Google search was checked to ensure it met the inclusion criteria. Only records that met the criteria were downloaded and extracted. The Google media search was conducted on three days in July 2021 (1st, 14th and 28th), then updated on September 9 2021, to capture additional articles from late July 2021, and then updated on April 26 2022 to identify additional articles specifically on the Pan American and Asian Games. The dates for inclusion were from January 2000 up until 31st July 2021.

The authors assessed the risk of selection bias, reflecting on the first author’s extensive publications on this topic. Therefore, to minimise reviewer selection bias, the search strategy followed a rigorous systematic review process that searched for a broad range of evidence including grey literature and unpublished reports, with dual review of the citations against the inclusion and exclusion criteria by the authors [44], the second author of whom has only one published citation on this topic.

### Data Extraction, Mapping and Analysis

Data were extracted using a form specifically designed for this study. The year, citation (title, author, source), aim or purpose, the event, timing (pre or post), type of evidence, country of author, target audience and a summary of the outcomes were extracted for all sources. Some additional information was extracted relevant to the type of source (e.g., methods and sample for primary research) that benefited the interpretation and relevance of the outcomes. To minimise bias in data extraction and analysis by the first and second authors, research assistants (health profession graduates) independently extracted data from each source, and this was then cross-checked by the first and second authors. To analyse the outcomes from each source, the researchers followed an iterative process where emerging narratives were discussed, recorded and refined as data were extracted. The questioning by the independent research assistants who had not had experience with this setting helped the authors reflect on the content and meaning of each source. The data were grouped according to the specific event and presented chronologically to give a perspective on the evolution of the development of food provision and nutrition support. In the synthesis phase, the data set and narratives were reviewed by the researchers to identify the overarching themes. The emerging themes were discussed with experts who had previous experience of food provision at these events.

## Results

The search of the databases using the specified search terms identified 2821 pieces of evidence relevant to food, catering and nutrition specific to the Olympic Games or other mega events. After duplicates were removed, the titles and abstracts of all remaining articles were reviewed to establish if they met the inclusion criteria, leaving 170 articles eligible for inclusion. Full texts of the remaining articles were obtained and reviewed for relevance to the food provision at the event. Of these 86 were excluded as they did not meet the criteria of a mega/major event, were about catering for the public or outside of the event or were about athletes’ diet but not specific to the village or venues.

**Fig. 1:**
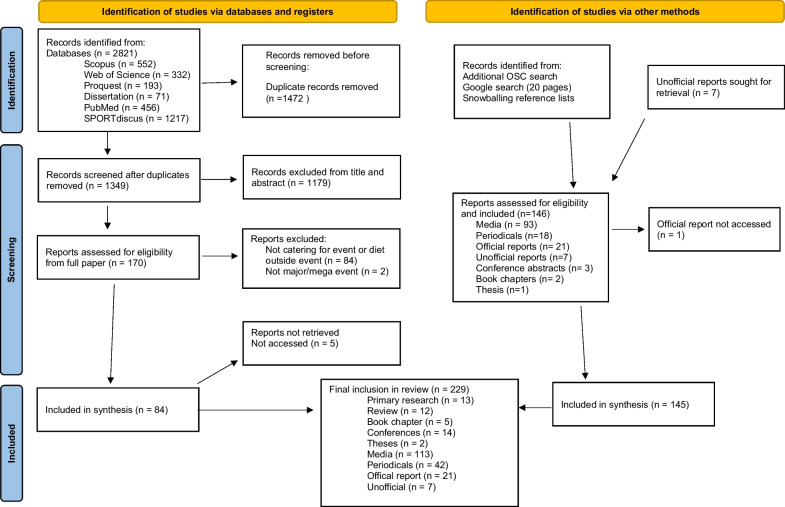
PRISMA 2020 flow diagram of the data source selection process [58]

**Fig. 2:**
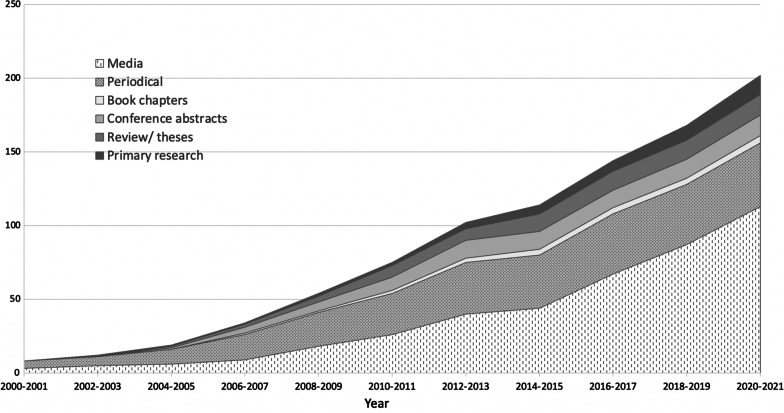
Growth in types of evidence over time

**Fig. 3:**
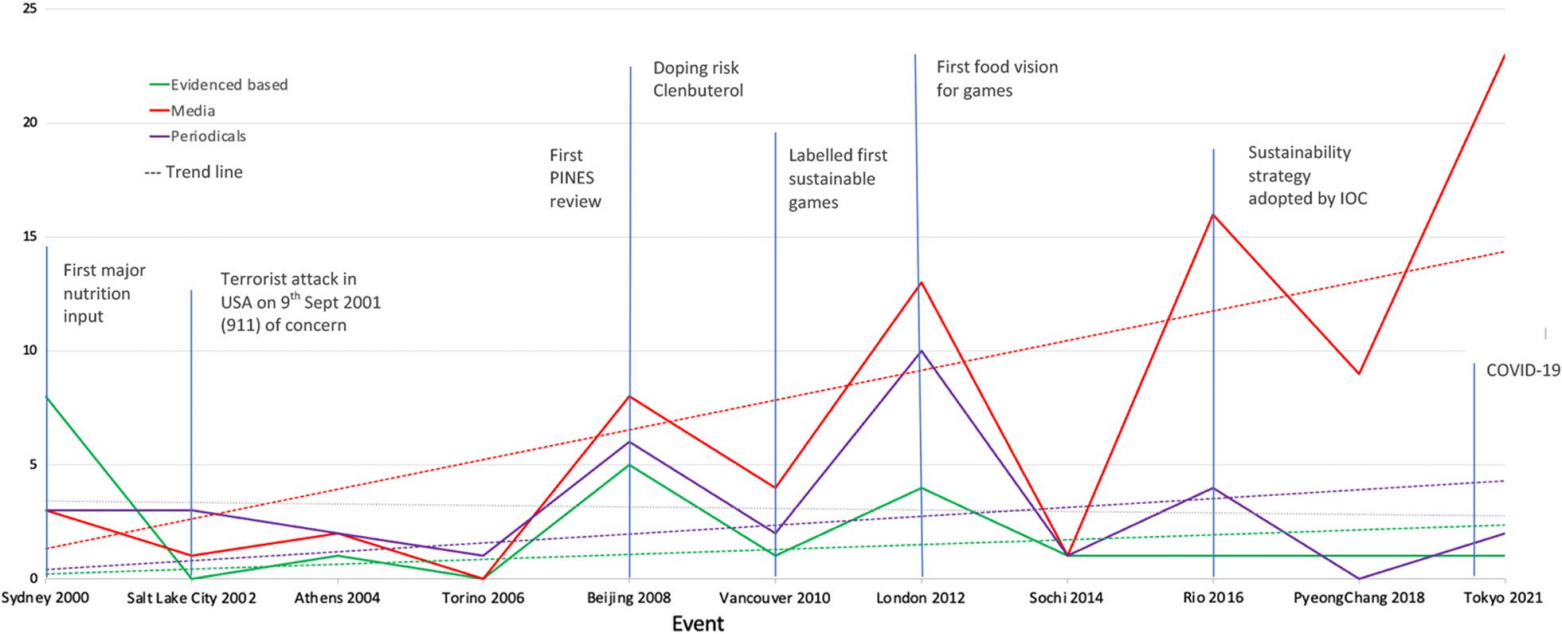
Data sources for Olympic and Paralympic Games 2000–2021

The search of the Olympic Studies Centre identified reports for all OPG post 2000 [45–52], except for the Rio 2016 Summer OG. A report for the Melbourne 2006 Commonwealth Games was also unable to be accessed. Other relevant documents identified included food visions for the London [53], Rio [54], and Tokyo OPG [55], the London 2012 Food Legacy [56] and the IOC Sustainability Strategy [57]. An additional article for the periodical Olympic review was identified. Seven unofficial reports were identified through snowballing and the first author's own sources. The Google search identified an additional 60 Olympic and Paralympic Games, 9 Universiade, 7 Asian Games, 6 Commonwealth Games, and 6 Pan American Games media articles. The final 229 sources for inclusion were 13 primary research studies, 12 reviews, 14 conference abstracts/papers, 5 book chapters, 42 periodicals, 113 media articles/reports, 2 theses, 21 official reports and 7 menu reviews. Five relevant periodicals could not be sourced. The flow diagram is presented in Fig. 1. There has been substantial growth in evidence, in particular media reports, over time (Fig. 2), although there is variability in the type of evidence reported for each event (Fig. 3).

### Meta-narratives

There were 11 narratives that emerged from the data extraction phase: ‘description of meals, menus and food’, ‘vision of the food provision’, ‘food safety’, ‘catering company involvement’, ‘sponsorship or contracts with food companies’, ‘athlete perspective’, ‘stakeholder perspective’ ‘athlete food intake,’ ‘nutrition input in food provision’, ‘food environment’ and ‘sustainability’ The description for each narrative is provided in Table 1.

**Table 1:** Description of meta-narratives

	Narrative	Descriptor
1	Description of meals, menus and food	Details of the menu itself including quantities (both meals and ingredients), the menu cycle, menu items, cuisine options, cultural aspects of menu, function of the menu and meal preparation and production,
2	Vision of food provision	Vision for the provision of food for the entirety of the Games or lasting effect post-Games
3	Food safety	All aspects of food safety related to general food safety concerns and to minimising contamination and risk of foodborne disease, doping, and allergens
4	Catering Company Involvement	Information about the catering company, chefs, workload, experience, staffing
5	Sponsorship or contracts with food companies	Details of sponsors providing food at the competition, their input or impact of their involvement
6	Athlete perspective	Athletes’ opinions on the food, catering services, dining hall experience, food preferences, and food choice
7	Athlete food intake	Athletes’ dietary intake or diet quality from menu, quantities consumed
8	Stakeholder perspective	Perspective of dietitians, coaches, organisers, caterers, chefs and other interested parties about the food, menu and catering services
9	Nutrition input in food provision	Dietitian involvement in the provision of the food service (for example, nutrition, labelling, working with caterer, nutrition service to athletes)
10	Food environment	The specific eating environment, information on the type of food service system, meal distribution and service models, procurement and delivery of food
11	Sustainability	Environmental sustainability in the context of food provision such as: plans specifically prior to, during or post-Games, vision of sustainability practices, awareness of sustainability by stakeholders, sustainable food waste and non-food waste (paper and packaging) management associated with food production and meal service

The majority of primary research studies (*n* = 12, 92%), conference papers (*n* = 13, 93%) and book chapters (*n* = 5, 100%) were predominately from Australia and focused on nutrition input in food provision or stakeholder perspective, and review articles from China (*n* = 6, 50%) with a focus on food safety. Most periodicals (*n *= 28, 66.7%) were targeted at the foodservice industry while media was targeted at the general public (*n* = 48, 42%) and foodservice industry (*n* = 49, 43%), followed by athletes and teams, and government bodies, and mainly focused on the description of meals, the food environment, catering company involvement, food safety and stakeholder perspective. The official documents described the food delivery, provided a vision of the food provision or mentioned sustainability. The evidence type for each narrative is shown in Table 2.

**Table 2:** Record type per meta narrative

Meta-narrative	Evidence based records					Grey records			Total
	Total evidence based *n* (%)	Peer reviewed primary research	Peer reviewed reviews/theses	Book chapters	Conference abstracts	Total grey *n* (%)	Periodicals	Media	*n* (%)
Description of meals and menus	18 (14)	2	6	5	5	109 (20)	24	85	127 (19)
Vision of food provision	5 (4)	0	2	1	2	28 (5)	7	21	33 (5)
Food safety	16 (13)	3	9	3	1	52 (10)	15	37	68 (10)
Catering company involvement	15 (12)	3	5	4	3	69 (13)	18	51	84 (13)
Sponsorship/ contract (food companies)	4 (3)	0	1	2	1	47 (9)	13	34	51 (8)
Athlete perspective	13 (11)	5	1	1	6	26 (5)	3	23	39 (6)
Athlete food intake	5 (4)	2	1	1	1	17 (3)	4	13	22 (3)
Stakeholder perspective	13 (11)	5	3	0	5	49 (9)	18	31	62 (9)
Nutrition input in food provision	23 (19)	7	3	5	8	24 (5)	12	12	47 (7)
Food environment	8 (7)	1	3	3	1	76 (14)	19	57	84 (14)
Sustainability	3 (2)	1	1	1	0	37 (7)	8	29	40 (6)
TOTAL	123 (100)	29	35	26	33	534 (100)	141	393	657 (100)

### Evidence of Food Provision at the Olympic and Paralympic Games

All sources tend to consistently report on the diversity and size of the food provision, with reference to the range of cultures, numbers of meals produced, the quantities of foods used across the event, the service delivery and the menu cycle. The official reports of the Olympic Games, where available, provide some information on food service delivery and may have areas of focus, such as food safety for Beijing 2008 [48] or sustainability for the London 2012 OPG [50]. However, the narrative from the official reports post-Games editions was generally focused on a basic qualitative and quantitative description of the meals and menus (such as number and type of food outlets and meals served over the course of the event), rather than providing insight on the outcomes of the food provision. No official report could be located for the Rio 2016 OG. The only mention of food provision relevant to the Paralympic Games was in reference to lowering food counters to allow access for athletes in wheelchairs at the PyeongChang 2018 Winter OPG [52]. Since London 2012, there have also been pre-Games published reports by the Organising Committee on the food vision for the event. These have predominately focused on addressing sustainability with the food provision and more recently have aligned with the IOC sustainability strategy [57].

The narrative of the primary research studies that have been conducted on this topic focused on food safety or the perspective of different stakeholders such as athletes or nutrition experts and provided more insight on the positives and issues related to the food provision at each event. The main narrative for each event post Sydney 2000 OG is provided in Table 3. This provides a follow on from the food provision at the summer Olympic Games provided in the previous historical review [5]. It is apparent that while the evidence has expanded over time (Fig. 2), there has been more focus on the summer Olympic Games than the winter Olympic Games (Fig. 3), which is reflective of the number of participants and countries at these events. There has been minimal evidence on the food provision at the Paralympic Games over this time.

**Table 3:**
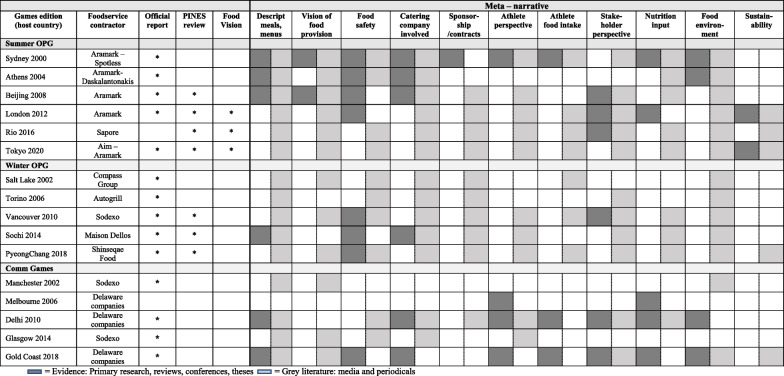
Record type per major event and per narrative (evidence-based or grey records)

Since the 2008 Beijing Games and as outlined in the historical review [5], the menu for both the summer and winter OG has been reviewed by an external group of sports nutrition professionals (PINES—Professionals in Nutrition for Exercise and Sport—https://pinesnutrition.org/). This has resulted in a series of seven unpublished reports to the International Olympic Committee (IOC) and two peer-reviewed publications that provide expert review of the food provision at the London 2012 [59] and Rio 2016 [60] Summer OG. An interview conducted by Burke (2009) [61] also provides insight into the roles of a dietitian catering at the Beijing OG. The narrative from these reports has predominately been focused on expert opinion and nutrition input into food provision. A summary of the events with official reports, PINES reports and caterer details has been provided in Table 3. The PINES reports on the menu reviews and the research articles provide more detail on aspects of the foodservice delivery and suggestions for improvement. It is apparent that particular editions (Rio 2016 and Sochi 2014) identified a number of issues with the proposed foodservice prior to the Games (unpublished reports) and in the case of Rio, significant issues during the event that included a lack of signage, labelling of menu items as well as the food itself [60]. The editions with more positive feedback include the London 2012 Summer OPG [59, 60, 62], Vancouver 2010 and PyeongChang 2018 (Professionals in Nutrition for Exercise and Sport (PINES) unpublished reports) Winter OG. Despite these disparities between events, there have been some consistent issues raised through the review process. These include the provision of gluten-free items, dairy items, snack foods, sports foods, high-energy drinks and provision for those in weight category sports. The summary feedback from these reports has been provided in Additional file 1.

### Evidence from Similar Major Competitions

There is more evidence from other major competitions compared to the Olympic Games, which is most likely due to the high stakes and political nature of OGs. Reports from similar major competitions focus on the food environment. Since Sydney 2000, evidence on the outcomes of an integrated food and nutrition program has expanded to other competition events, in particular the Commonwealth Games from 2006 to 2018. A summary of both published literature and unpublished reports from the Commonwealth Games have been provided in Additional file 1. The evidence comes from predominately observational studies that have looked at athletes’ and diners’ experiences of the food provided. These studies have demonstrated that both athletes and officials value access to a nutrition service and will seek menu and food advice to assist them in planning a diet to meet their specific needs when a comprehensive nutrition program is available at major events, and this extends beyond performance nutrition [63]. There are also reports on the dining experience of athletes and their use of a nutrition service [64–66], their reported dietary intake in this environment [67], the use of nutrition labelling [68] and their food choices [2, 4, 69, 70].

### Role of Media and Periodicals

Similar to peer-reviewed literature on this topic, the majority of media reports and periodicals focus on food provision at the summer Olympic Games, although there is some reference to food provision at the winter Olympic Games, Commonwealth Games, Asian Games, Pan America Games and Universiade. There was only one media article specifically relevant to the Paralympic Games. However, unlike official reports and structured research, some of the grey literature reports on more controversial aspects of the food provision that adds value to the narrative of the topic. Relevant quotes in the media from key stakeholders have been provided in Additional file 1. Many articles reported on the role of the catering company, particularly their experience at previous events with an emphasis on the large scale and “unique” nature of catering at mega Games or the “once in a lifetime” opportunity for catering staff to experience such events. Media articles commonly reported on general interest stories about what the athlete will experience in the dining hall at the athletes’ village and showcasing the facilities and array of meal options, or the role of sponsors. Food safety was also featured in the grey literature, in particular controversy around contamination of animal products in Beijing and in Tokyo post the Fukushima accident. More recent media focused on the delay of the 2020 Tokyo OPG and the impact of COVID-19. As demonstrated in Fig. 1, the number of media and periodicals on this topic have grown substantially, most likely due to the ease of adding and viewing online content. We note that social media content on sites such as Instagram, Facebook, YouTube and Twitter would provide additional current commentary, particularly from the athlete perspective; however these were not included in this review. Current data suggest that there has been an exponential growth in the use of social media (5% of US adults in 2007; 79% in 2019) [71] and therefore this would be relevant to the most recent major events.

## Synthesis and Discussion

The narrative synthesis of past and current issues at these events based on the evidence is presented below in the context of the broader literature. While there were 11 meta-narratives that emerged from the data extraction phase, the synthesis of this evidence resulted in four major themes which are discussed below. Future challenges with suitable food provision in this environment are also discussed.

### Scope, Experience of Caterers, Role of Sponsorship and Outcomes of Food Provision

Through most sources (media, periodicals, research and PINES reports) there was recognition of the unusual nature of these events and the importance of the contracted caterer in understanding the scope. Those events with large scale international caterers (Aramark, Sodexo, Delaware) received more positive reports from experts. However, this was not the case for those that contracted local caterers to provide the Olympic village catering. The comparison of feedback from Rio [60] and London [59] papers suggests that the lack of consistency in food provision between events is indicative of the caterers' experience. This has also led to commentary about the need for much earlier involvement of nutrition expertise in catering to help guide the development of the menu to suit all attendees [72]. The Sochi 2014 Winter OG were labelled as the ‘Hunger Games’ due to the lack of variety of food for athletes [73] and this was supported by concern raised about the menu in the pre-Games  PINES report [74]. Data presented in this report from experts rated the variety of foods, in particular providing for the range of cultures and different dietary needs such as vegetarian and vegan as poor. This is supported by a periodical article from the USA that reported on the dining hall at the Sochi 2014 OG. This mentions the lack of information on ingredients in the food, which was ‘forbidden’ to be disclosed according to the head of the food program [75]. The Rio 2016 OG Organising Committee produced a food vision that was published in advance of the event [54], but there were ongoing issues with lack of caterer experience apparent which meant that strategies that could have helped to better manage food waste were overlooked [75]. Collectively, it appears that a suitable caterer appointed early to manage the food provision is critical to success.

The independent PINES reports add value to the process of feedback to the caterer, particularly if there is a  review on paper and on site as per London 2012 [59]. An additional challenge is the consistent delivery of food across multiple villages. The use of different athlete villages is not uncommon for the winter Olympic Games where competition venues can be geographically far apart. The first mention of separate villages occurred in the 2007 Winter OPG in Torino [47] where the challenges of transportation and altitude conditions were mentioned. While ‘food to go’ has been available to be ordered by athletes at a number events over the past 20 years, ‘Grab and Go’ food carts with readily available food are a newer approach to assist athletes who do not have time to go to the main dining hall [50] More recent PINES reports also provide more detailed assessment of multiple villages, food outlets and venues, in addition to ‘food to go’ and can provide a sense of areas that create ongoing challenges for caterers. For example, improvement in the variety of food provided was a key feature of the 2018 PyeongChang Winter OPG [76].

Sponsorship by companies such as Coca-Cola™ and McDonalds™ was reported as having both a positive [77] and negative [78, 79] impact on food provision, dependent on the type of evidence. In the media, vested interests voiced their concern that sponsorship contracts restrict local food suppliers from showcasing their products and the opportunity to make an impact or highlight criticisms from health organisations of the relationship between fast food sponsorship and the OG [80]. The media also reported on the athletes’ perspective of the suitability of the food and the impact of fast-food chains such as McDonalds. For example, Jamaican sprinter Usain Bolt reportedly ate chicken nuggets from McDonalds throughout the 2008 Olympic Games because he did not trust the food in China [81].

The various sources of evidence considered the cultural diversity of the menu. As food choice of athletes and others can be determined by cultural norms and religious beliefs [64], a key principle of menu planning is to consider culture and religion to ensure a menu is acceptable to the consumer. Menu reviews by PINES experts from different countries evaluate the appropriateness of Games menus to meet the diverse cultural needs of athletes. Athletes appear to want representative items on the menu from their own culture [64] but also embrace the opportunity to taste different foods and cuisines [82, 83]. Grey literature, in particular, mentions the menu as a means to showcase the hosting country’s cuisine [84]. However, the nature of the menu is reported to be influenced by Organising Committees, based on the role they want the menu to represent. This may be to establish the proportion of Western food to local cuisines [85]. Meeting the cultural needs of athletes can be difficult if caterers have not been exposed to different cuisines [86]. A larger scale catering company with a history of catering at several Olympic Games such as Aramark Corporation can bring in chefs from various nations who learn from each other, thus being exposed to different cuisines and cultures [87]. Pelly and Tweedie [72] found that although caterers focus on athlete satisfaction of the menu from a cultural perspective, this is difficult to achieve from one event to the next in different countries and that cost, the environment and chef creativity ultimately influence the menu design.

### Food Safety, Security and Risk of Illness

Food safety and security have been a focus across all sources of evidence for the past 20 years, and for most events has been about mitigating risk beforehand rather than reporting on actual incidences. Initial concerns focused on security post the terrorist attack in the USA in 2001, which was particularly relevant to the Salt Lake City 2002 Winter OPG. One media article mentioned mandatory FBI checks of catering supplies [88] and the official report discussed fencing, security guards and surveillance cameras [45].

Concerns were raised prior to the Beijing 2008 Games around inadvertent doping with clenbuterol in Chinese meat [89] leading to the specific focus on food safety by the Government of China [90] and a specific food safety subcommittee of the Beijing Organising Committee of the Olympic Games (BOCOG) [91]. This led to the farm to table approach introduced in Beijing to allow for a track back system and the use of the Hazard Analysis Critical Control Point (HACCP), and along with a public health campaign, prevented any foodborne illness during the event [91]. The resulting recommendations from Beijing were that adequate resourcing for legislation, laboratory testing, inspection, and training of food handlers [91] was paramount for all major events. Despite this, there were reports that food safety was less than adequate due to differences in what is considered safe practice in non-Western countries [91].

The mitigation of risk of disease outbreaks and the relationship to food safety at major events has been highlighted at previous events [92]. Subsequently, a paper that discussed improving health services at the Olympic Games identified that gastrointestinal and foodborne illnesses were the most common communicable diseases, although uncommon [93]. Foodborne illness includes human norovirus, hepatitis A, hepatitis E, rotavirus and astrovirus. An outbreak of norovirus before the 2018 PyeongChang Winter OG was reported in the 2018 official report [52]. This resulted in a response that led to the use of sanitizers, face masks and quarantine measures during the event. In 2018, the potential health risks for the 2020 Tokyo OPG, including a range of vaccine-preventable diseases, communicable respiratory diseases, food and waterborne illness and heat-related illness, were highlighted [94]. This recommended that hygiene measures needed to be encouraged.

#### COVID-19 and the Impact on Food Provision

The outbreak of the Novel Coronavirus SARS-CoV-2 (COVID-19) that occurred in Wuhan, China in 2019 [95] was not anticipated, nor was the postponement of the Tokyo 2020 Olympic and Paralympic Games to 2021. The environment of the village dining hall, where 4000 or more people consumed food in close proximity to one another, was considered a significant risk for the spread of COVID-19. Strategies that appear to mitigate transmission of COVID-19 are personal hygiene, washing hands and social distancing, although evidence of transmission by food is less apparent. Reducing the frequency of touching surfaces and disinfecting can also reduce transmission [96]. The impact of COVID-19 resulted in changes to the food delivery at the Tokyo OG with the introduction of plexiglass dividers, gloves and limits on the number of people attending the dining hall [97, 98]. Although these COVID countermeasures were described in the media [99], it was interesting that food safety concerns were more to do with the fear of food from Fukushima due to radiation than COVID-19 [100]. However, the fear of COVID spread, in combination with the fear of radiation contamination of food, influenced some countries to provide their own food program and catering provisions to their athletes and team delegation at the Tokyo OG [98]. For athletes, concerns were raised over the impact on social interaction in the village [99].

There is likely to be a delay before evidence-based literature emerges on the impact of food provision on prevention of COVID-19 illness, but it is apparent that future changes to the way food is delivered will remain as was evident at the 2022 Beijing Winter OG [101]. Furthermore, the impact of COVID-19 on restricting large scale catering, the global food supply and shorter stay protocols have potential longer term impact on food provision at future Olympic Games. Subsequent to this review, the Birmingham 2022 Commonwealth Games housed athletes in three villages limited to no more than 3000 athletes due to the impact of COVID-19 [102]. A recent study that investigated stakeholder perspective of the impact of COVID-19 suggested that consistent guidelines and support from organising committees are needed for caterers to ensure safe food provision and environments [103].

### The Role of Nutrition in Food Provision

The importance of nutrition expertise in food provision was a common theme that emerged from the narratives, particularly in the primary research papers and the PINES reports which focused on reporting different stakeholder perspectives with summaries of areas of improvement and nutrition labelling of menu items. There were also reports of dietitians' input into the menu, the presence of a nutrition kiosk located in the village and nutrition labelling of menu items at certain events. There appeared to be a disparity between the perception of the success of certain events based on the source of information. Official Organising Committees reports rarely reported on nutrition input and were generally positive; however papers that reported nutrition experts’, caterers’ or athletes’ perceptions focused on areas of improvement.

#### Therapeutic Diets and Dietary Trends

One of the reported challenges is the increased demand in therapeutic dietary needs, particularly food allergies or intolerances. These may range from peanut/tree nut, seafood, wheat, gluten or lactose to avoidance of highly specific food chemicals (e.g. salicylates, amines, food preservatives or additives) that occur widely in the modern food supply. There was evidence of a substantial increase in requests for gluten-free foods at these events with around 13% of elite athletes reporting to follow a dietary regimen related to a specific food allergy or intolerance (most being low-lactose or wheat-free) [34]. There has also been reported a wide range of clinical conditions where diet is central to clinical management (e.g. diabetes, coeliac or cardiovascular disease, inflammatory or irritable bowel syndrome). This appears to have been reported more commonly in evidence-based sources that are driven by nutrition experts but is of less interest to the media.

Increase in the diagnosis of dietary issues as well as trends in self prescribed diets by athletes (unpublished data by the first author, Commonwealth Games 2018) has led to a demand for a menu and food delivery that enables diners to manage these conditions with ease and confidence. In addition to these clinical issues is an increased demand for tailored, personal food preferences such as vegan or vegetarian diets and preservative free or natural/organic food (unpublished data by the first author, Commonwealth Games 2018). Specific food and ingredient avoidance has been reported as a means of avoiding gastrointestinal distress in athletes [36]. The identification of allergen labelling was recommended in advance of the Beijing OG [104] and was also suggested as important by athletes attending these events [68].

#### Variability in Sports Nutrition Service

It is apparent from the evidence from the nutrition kiosk that Olympic teams and individual sports have varying degrees of nutrition support and understanding [35]. Sports nutrition experts in Western countries are usually registered dietitians with specialised clinical dietetic and sports nutrition training, whereas in less well-developed countries, nutrition advice may come from those with limited education in clinical or sports nutrition, often those with medical or some other type of allied health training where nutrition education is a minor (if addressed at all) part of professional education. Historically, dietitians/nutritionists have been less frequent members of the sport science support teams at competition events, with nutrition support provided in advance of major competition events. Limited within-team nutrition and dietetic expertise for many countries was documented at the Sydney 2000 OG [32], but there has been minimal research on the level of expertise and degree of involvement of sports dietitians/nutritionists within OG teams post Sydney. Evidence to support a nutrition kiosk within the dining hall of Olympic or Paralympic Games comes from other international events such as the 2006 [105] and 2010 [63] Commonwealth Games and the 2017 Universiade [35]. The frequent use of this service indicates that it is an important professional resource when this is not part of the support provided within teams. Empirical evidence from these events suggests that nutrition labelling, exposure to different cultural and therapeutic foods, provision of dietary plans for home and education sessions held with coaches and teams were of great value to those accessing these services [35, 63, 105]. Furthermore, those seeking advice tend to have dietary intakes that are less than adequate for their general health, let alone for sports performance [35, 67]. The food environment at the OG has the potential to influence and educate many stakeholders on the importance of nutrition for both health and performance and provide resources and guidance to upskill the wider sports science community. This is an important but currently intangible legacy of OG participation. Furthermore, the OG has previously been scheduled during Ramadan which has resulted in a focus on the impact of fasting on sports performance [106].

Recently, stakeholders’ perspectives on the integration of nutrition in catering was investigated to better understand the barriers and enablers to improving the food provision at major events [72]. The logic model presented in this study demonstrated that the barrier to change is at the level of the organising committee, but this is significantly influenced by the local environment [72]. The collective evidence from all the sources suggests that the integration of sports nutrition expertise into the planning and operational stage of these events has better outcomes in terms of food provision that is appropriate for athletes’ current needs.

### The Global Increased Focus on Environmental Sustainability in Relation to Large Scale Events

Concern about environmental sustainability has also increased for organising committees and caterers with a focus on the use of the whole animals and a reduction of food packaging [53, 55]. This aligns with the timely and topical release of the EAT-Lancet Commission on healthy diets from sustainable food systems [39], which advocates the need for sustainable food systems that provide health benefits but also sustain a healthy planet. The IOC has recently produced a Sustainability Strategy [57] and Olympic Games Guide on Sustainable Sourcing [107] in response to global trends and is part of their strategy for change at the Olympic Games [108]. These reports highlight food and beverage services as a high priority area in improving sustainable practices that should be factored into tenders for the OPG. Clearly, the production of large volumes of food produces significant waste and the focus for the IOC is on reducing surplus and spoilage, and redistribution of excess food to the workforce, the community or to animal feed. Data collection on food waste is emphasised as important in this report, but interestingly no data on food waste have been reported in the literature. Examples from media particularly focused on Rio 2016 and the social justice initiative by celebrity chefs to collect and use food waste from the OG village, to provide meals to the poor and to highlight the worldwide issue of food insecurity. Although the chefs behind the program reported that there was support from the Rio 2016 Organising Committee and IOC, they also reported that they were initially met with disinterest [75]. Other imperatives particularly relevant to food provision are consideration of animal welfare and biodiversity, packaging, sustainable fish and shellfish farming, and water use [109]. Interestingly, a review of the sustainability of the OGs suggests that they have become less sustainable over time, and this applies to the editions that proclaim to be sustainable Games [109]. Those that rated worse in sustainability scores were the Rio 2016 and Sochi 2014 Winter Games, which aligns with poorer feedback on the food provision due to lack of experience of caterers. Despite certain events such as London 2012 OPG having a strong focus on sustainability in their food vision [53], not all proceeded as planned. An article on the London 2012 OPG suggests that the Olympic connection to sustainable local practice was limited and that multinational caterers were more likely to provide food than local suppliers [110]. During the event there was a media report of whistle-blowers talking about the large amount of food waste in the village and post the event which led to an investigation by the Games environmental watchdog [111].

Both the Tokyo 2020 strategy for food and beverage [55] published in 2018 and a 2020 review of the sustainable resource management for the Tokyo 2020 OPG [98] suggest that there was a large emphasis on reducing food waste at this event. However, changes in food delivery due to COVID-19 (such as cleaning, use of gloves and packaging) have changed the focus to food safety regardless of sustainability goals. Suggestions that the Olympic Games must downsize to reduce resource requirements and be rotated amongst the same cities [109] would have an impact on sustainability. Equally, the management of food procurement and waste with the continued use of experienced caterers would also benefit sustainability outcomes.

## Future Directions

### The Legacy of the Food Environment

There is limited literature specifically relevant to the broader food environment and the influence of the OG food environment on athletes, teams, caterers, professionals and the public. The recently published 'Determinants of Nutrition and Eating' (DONE) framework [112] provides a review of the multi-disciplinary literature on determinants of food choice. These are mapped into four broad categories: individual, interpersonal, environmental and policy. This paper refers to the lack of research on the influence policy has on changing eating behaviours. There is current interest in the individual and interpersonal determinants of food choice in athletes [2–4, 69, 70], as well as the impact of food provision on promoting the health of spectators [113], but no literature on the influence of policy on the OG food environment and how this may have broader reaching influence than just the period of the Olympic Games. The recently proposed foodservice systems model [72] identifies that food provision issues occur predominately during the early planning phase due to a lack of continuity between one event and the next. The reactionary response to issues and the lack of budget to support change must be addressed in the current climate for the ongoing sustainability of food provision at these events.

Nevertheless, the provision of food in this environment has considerably more to offer than just performance. Athletes and team officials are immersed in this dining environment accessing the village dining hall three times a day or more over 2–3 weeks. Diners gain first-hand experience of different cultural, religious and dietetic/therapeutic aspects of food and are exposed to the diversity of the OPG menu. Patrons share the communal dining area with not only teammates but also teams from around the world. This brings people together and helps build respect, understanding and social connection outside the sporting arena. This shared dining environment is a positive part of the OPG experience.

Legacy planning is a key component of the IOC Olympic agenda 2020 [114]. Potential intangible legacies resulting from the food provided at the Olympic and Paralympic Games include the development of nutrition expertise (globally and locally), as well as the broader influence of the food environment on cultural competence and efforts in sustainable practices by caterers and understanding of specific sports nutrition requirements of athletes as a dynamically changing science. This will help identify the need for ongoing education in food and nutrition, especially for those working in countries with less professional expertise in this specialised area.

In the current global environment, there is likely to be substantial change in knowledge and development in food and nutrition, particularly in relation to broader issues such as sustainability, and thus continuity from one event to the next is critical. To assist with overall governance and consistency of food provision at these events, development of a food and nutrition framework that is integrated into the food vision and subsequent catering policy would be of benefit. This could address contemporary areas of concern such as safe food and doping, cultural suitability, nutritional content and dietary trends, suitability for sports performance, sustainability and waste management, and procurement of food.

## Limitations

A limitation of this study was the restriction to articles published in English as OPG occur in countries where English may not be the first language, and thus limited evidence may come from these events. Another limitation is that comprehensive reports on food provision by the catering companies contracted at each event were not included as this is commercial in confidence material not accessible by the research team. Further evidence to support this review could be obtained from interviews with a broader range of stakeholders with experience in food provision and nutrition support at mega events. Future perspectives such as understanding the impact of COVID-19 on catering at mega events from those with past experience will allow for the development of relevant policies that can be incorporated into tenders and be written up as Olympic Games Guides, similar to the current guide on sustainability. With the advent of social media, it is feasible that some information relevant to this meta-narrative may have been overlooked for the most recent events. However, as the narrative triangulated across peer-reviewed sources, official reports and the grey literature, it is unlikely that this would have significantly impacted the outcome of this review.

## Conclusion

The synthesis of evidence in this review has demonstrated that key global events have been a driver for food provision and impacted the food environment at major events. The food vision and the resulting changes to food delivery will be of interest in the future, particularly in the context of pandemics and climate change. Further research on the Paralympic and winter Games will provide more contemporary evidence for the role of food provision in varied settings with diverse populations. The study of environmental influences on nutrition and eating behaviours is a growing area of interest both in performance nutrition and public health due to the strong links between dietary intake and chronic disease, exercise recovery and optimal performance.

The OPG is a unique food environment where the vast availability of easily accessible and culturally diverse food allows exposure and interaction with different cultures and eating styles. It has a substantial capacity to positively influence dietary choice and build cultural competence and social inclusion. To date, this important legacy of the OPG has not been explored. The food environment at the OPG is a potential example of ongoing excellence in food provision, not only in quality and food safety, but provision of a sustainable food supply that will optimise and educate on health and performance and be a living example to organisers, athletes, teams, caterers, and the sport and nutrition community of the value and role food has in society.

## Supplementary Information

**Additional file 1**. Spreadsheet of extracted data from all sources.

## Data Availability

Extracted data from all records in this review have been provided in the supplementary spreadsheet that accompanies the review.

## Abbreviations

BOCOG: Beijing Organising Committee of the Olympic Games
DONE: Determinants of Nutrition and Eating
HACCP: Hazard Analysis Critical Control Point
IOC: International Olympic Committee
OPG: Olympic and Paralympic Games
OG: Olympic Games
PINES: Professionals in Nutrition for Exercise and Sport
RAMESES: Realist And Meta-narrative Evidence Syntheses: Evolving Standards
